# A familial case of cleidocranial dysostosis presenting upper limb ischemia

**DOI:** 10.1590/S1516-31802005000600009

**Published:** 2005-11-01

**Authors:** Walter Campos, Roberta Murasaki Cardoso, Ronald Fidelis, Erasmo Simão da Silva, Rodrigo Ramos

**Keywords:** Cleidocranial dysplasia, Cranio-facial dysostosis, Dysostosis, Mandibulofacial dysostosis, Displasia cleidocraniana, Disostose craniofacial, Disostose, Disostose mandíbulofacial

## Abstract

**CONTEXT:**

Upper limb ischemia is not as common as lower limb ischemia but may cause severe impairment or disability if it is misdiagnosed.

**CASE REPORT:**

A case of a woman with cleidocranial dysostosis resulting in upper right limb ischemia is presented. This uncommon condition is an exceedingly rare cause of vascular compression that gives rise to thrombosis of the axillary-subclavian arteries. Only two cases have previously been reported.

## RESUMO

**CONTEXTO:**

A isquemia do membro superior não é tão comum quanto a isquemia do membro inferior, mas quando não diagnosticada ou diagnosticada de forma errada determina grave seqüela e até perda da extremidade.

**RELATO DE CASO:**

O caso de uma paciente com isquemia do membro superior, devida a disostose cleidocraniana, é apresentado. Esta é uma causa extremamente rara de compressão do segmento arterial subclávio-axilar que evoluiu para trombose arterial. Somente dois casos foram previamente relatados na literatura com esta condição e nesta forma de apresentação.

## INTRODUCTION

Acute upper limb ischemia is an uncommon condition, in comparison with acute lower limb ischemia. Arterial embolism, thrombosis of an atherosclerotic lesion and trauma are the principal etiological factors. Structural bone and muscle abnormalities such as cervical rib, exostosis of the first rib or clavicle, or fibrous bands may produce acute ischemia by means of mechanical damage to the axillary-subclavian arteries.

Cleidocranial dysostosis (CCD) is a congenital skeletal condition affecting membranous bones such as the clavicle and skull that may result in arterial compression and upper limb ischemia. The aim of this article was to describe a rare case of acute upper limb ischemia caused by cleidocranial dysostosis.

## CASE REPORT

**Figure 1:**
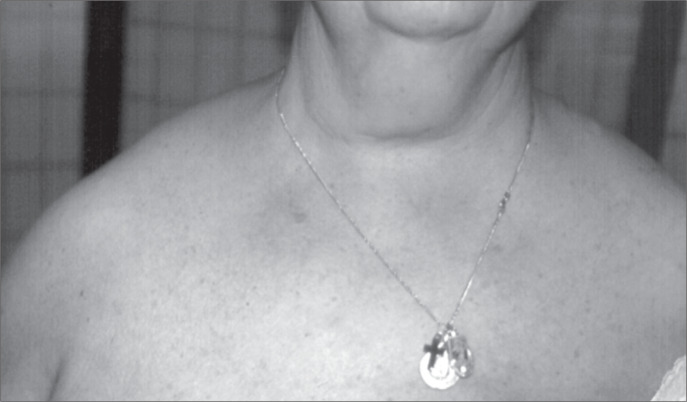
Photograph showing deformity of the upper thorax in a 65-year-old woman: appearance of drooping shoulders and elongated neck.

**Figure 2:**
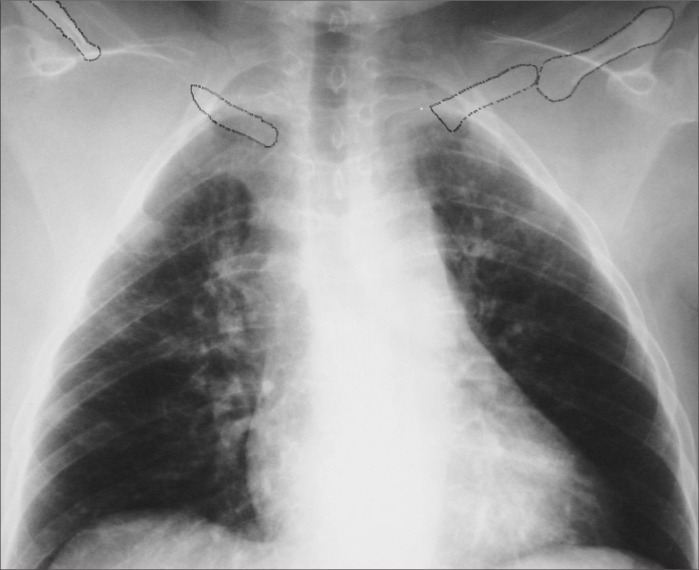
Posteroanterior view of chest radiograph showing the position of clavicular fragments in a woman with cleidocranial dysostosis.

A 65-year-old woman presented with a two-month history of pain in her right arm and forearm. She had also developed significant limitation of her activities but had not developed any finger ulceration. Her past medical history included left mastectomy due to breast cancer. Her mother and sister presented uni-lateral clavicle agenesis. Physical examination demonstrated prognathism (Figure 1), excessive mobility of the shoulders (double-jointedness), ischemia in the right hand, and no palpable axillary, brachial and radial pulses on the right side (the left side was normal). Chest x-ray demonstrated narrowing of the thorax and both hypoplastic clavicles (Figure 2).

**Figure 3:**
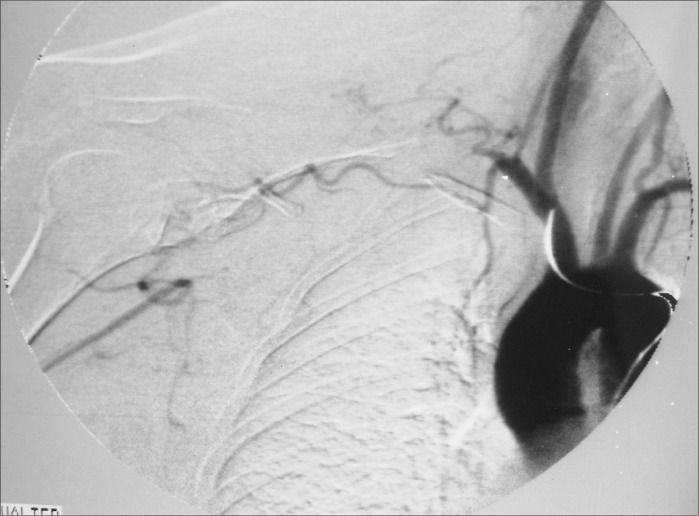
Arch aortogram of a woman presenting cleidocranial dysostosis showing occlusion of the axillary-subclavian arteries: contrast medium fills the brachial artery beyond the occlusion.

Color-flow duplex Doppler ultrasound scanning examination showed that the subclavian flow presented monophasic features. Digital angiography was performed and this displayed occlusion of the right axillary-subclavian arteries that spared the vertebral and internal thoracic arteries, with filling of the proximal brachial artery (Figure 3).

Due to the upper right limb limitation, angioplasty of the lesion was attempted through femoral access. It was not possible to cure the lesion using hydrophilic guide wire. The procedure was halted.

Open surgery was not attempted. Following the operation, the patient evolved without severe ischemia, and clinical management was carried out. She continued to have pain, but with some improvement following a course of physiotherapy, and the symptoms remained stable.

## DISCUSSION

CCD is a congenital skeletal abnormality with dominant autosomal inheritance, although some recessive forms have been described.^1^ The peculiar features of this disease result from faulty development of membranous bones, especially the clavicles and skull. The disease gene has been mapped to chromosome 6p21 within a region containing CBFA1, a transcription factor that activates osteoblast differentiation.^1,2^ Clavicular abnormalities give this disorder its characteristic appearance of drooping shoulders, an elongated neck and an ability to adduct the shoulders anteriorly (Figure 1). The commonest deformity is the absence of the central clavicular segment with small bony stumps attached to the sternum and acromion^3^ (Figure 2). In rare cases, the lateral portions or the entire bone is hypoplastic. Late closure of cranial sutures with Wormian bones and patent fontanels may occur, and there may also be late eruption of secondary dentition, zygomatic hypoplasia and underdevelopment of the jaw. Such patients seldom present neurological symptoms due to compression, and only two other cases of vascular damage to the upper limbs have already been reported.^3,4^

Short,^5^ in 1979, was the first to report a case of CCD causing upper limb ischemia and cerebrovascular symptoms related to this condition. It was of interest that the patient was a 63-year-old woman, who had both a hypoplastic clavicle and an ischemic right upper arm. An arteriogram showed proximal subclavian and vertebral thrombosis on the right side, and post-stenotic dilatation in the left subclavian artery, which was compressed between the first rib and a rudimentary clavicle fragment. Upon operation on both sides, it was found that the anatomy was grossly distorted and even upper right dorsal sympathectomy was impracticable. The wound was closed. On the left side, the hypoplastic proximal clavicle fragment was removed, without resection of the aneurysm.

In 1997, Qureshi et al.^6^ reported another case of CCD in a 63-year-old female patient who had upper right limb ischemic pain, resulting in the right hand being darker-colored and cooler than the left. Ulceration was present at the tip of the index finger and at the base of the little finger. Without palpable brachial and radial pulses, an arteriogram showed axillary occlusion beyond an abnormal clavicle, with no distal filling of vessels. An open-surgery repair was indicated, and, during the operation, dense scar tissue gave rise to technical difficulties in isolating and controlling the arteries. As in the earlier report,^5^ significant anatomical distortion and fibrosis were present. Abnormal tissue was found to be bonding the clavicle fragments. A polytetrafluoroethylene bypass was implanted. The patient was discharged with palpable distal pulse and healed hand ulcerations, while taking oral anticoagulant. Six months later, the bypass had occluded. No further intervention was attempted on the occlusion, despite the warfarin therapy, mainly because of the difficulties reported during the first operation. The ischemia did not appear to be threatening limb viability, and the patient was then treated clinically.

It is noticeable that the two previous reported cases and the present case all related to women in their seventh decade, which may indicate high gender and age-related prevalence for the symptoms.

In spite of the scarcity of experience reported in the literature, it seems to be evident that there are significant technical difficulties in performing open vascular repairs on this kind of arterial damage. The reported cases discouraged us from trying anything more aggressive in the case of our patient, since we were not dealing with limb-threatening ischemia. Although a long arterial segment was occluded, an endovascular approach was attempted in order to avoid the well-described anatomical and technical problems in the previous cases. As expected, it was not possible to cure the lesion, but the degree of ischemia did not worsen after the procedure.

## CONCLUSION

In spite of its rarity, cleidocranial dysplasia may lead to upper limb ischemia. A less aggressive approach seems to be reasonable, because of the significant anatomical distortions present in this condition.
